# Lecithin-cholesterol acyltransferase and paraoxonase-1 levels in atherosclerotic cardiovascular disease patients in Nigeria

**DOI:** 10.4102/ajlm.v13i1.2286

**Published:** 2024-07-17

**Authors:** Promise C. Nwaejigh, Maria O. Ebesunun, Oluwaseye M. Oladimeji

**Affiliations:** 1 Department of Chemical Pathology and Immunology, Faculty of Basic Medical Sciences, Olabisi Onabanjo University, Sagamu, Nigeria; 2 Department of Medicine, Lagos State University Teaching Hospital, Ikeja, Nigeria

**Keywords:** atherosclerotic cardiovascular disease, lecithin-cholesterol acyltransferase, paraoxonase-1, lipid profile, anthropometric variables

## Abstract

**Background:**

Recent evidence has linked changes in plasma lecithin-cholesterol acyltransferase (LCAT) and paraoxonase-1 (PON-1) levels with increased risk for development of premature atherosclerotic cardiovascular disease (ASCVD) in different populations. However, studies on this in Nigeria and sub-Saharan Africa are scarce.

**Objective:**

This study assessed the association between reduced plasma LCAT and PON-1 levels and an increased risk of ASCVD, and their potential as biomarkers for ASCVD.

**Methods:**

Atherosclerotic cardiovascular disease patients and healthy controls were randomly selected for this cross-sectional case-control study from the Lagos State University Teaching Hospital, Ikeja, Lagos, Nigeria, between March 2022 and March 2023. Plasma LCAT and PON-1 were determined by sandwich enzyme-linked immunosorbent assay, while the lipid profile was measured by spectrophotometry.

**Results:**

A total of 153 ASCVD patients (mean age: 52.92 ± 10.24 years) and 50 healthy controls (mean age: 46.96 ± 11.05 years) were included in the analyses. Stastistically significant increases were observed in the mean body weight, hip circumference, waist circumference, waist-to-hip ratio, body mass index, diastolic and systolic blood pressure (all *p* ≤ 0.001), and pulse rate (*p* = 0.003) compared to the control values. Statistically significant increases were also observed in the mean plasma total cholesterol, triglycerides, low-density lipoprotein cholesterol, and non-high-density lipoprotein cholesterol (all *p* ≤ 0.001). In contrast, the mean plasma high-density lipoprotein cholesterol, LCAT, and PON-1 (*p* ≤ 0.001) were notably reduced compared to the control values.

**Conclusion:**

The present study provides supportive evidence that changes in plasma LCAT and PON-1 could predispose individuals to risk of premature ASCVD.

**What this study adds:**

Plasma LCAT and PON-1 may serve as independent markers or complement other established cardiovascular disease markers to discriminate the risk of ASCVD when it is unclear.

## Introduction

Atherosclerotic cardiovascular disease (ASCVD) is characterised by plaque buildup in the arteries, leading to reduced blood flow and an increased risk of heart attacks and strokes.^1^ It poses a major health concern worldwide due to its high morbidity and mortality rates.^2^

Despite advances in treating ASCVD, identifying novel risk factors for ASCVD remains crucial for improving patient outcomes. In recent years, studies in Europe^3,4^ and Asia^5,6^ have focused on the role of plasma lecithin-cholesterol acyltransferase (LCAT) and paraoxonase-1 (PON-1) levels as potential biomarkers for predicting cardiovascular risk. However, there is a paucity of information on the relationship between these biomarkers (LCAT and PON-1) and ASCVD in Africa, especially in sub-Saharan Africa.

Paraoxonase-1 is an enzyme primarily synthesised in the liver and has been shown to have a protective effect against atherosclerosis,^7^ which is the underlying cause of several cardiovascular diseases, including ASCVD. Some studies have shown that individuals with low levels of PON-1 activity and concentrations have an increased risk of developing ASCVD, while others have found no association between decreased PON-1 activity and ASCVD.^6,8^

Reduced PON-1 concentrations in ASCVD patients suggest that PON-1 may play a protective role against the development of ASCVD. Low PON-1 concentrations have also been shown to be a predictor of ASCVD risk.^7^ In one study, individuals with low PON-1 activity were found to have a 2.4-fold increased risk of developing ASCVD.^9^

It is also important to note that the relationship between PON-1 activity and ASCVD is complex and multifactorial. Genetic variation, environmental factors, and lifestyle habits can also influence the regulation of PON-1 concentrations and, subsequently, ASCVD risks.^10,11^ For example, smoking has been shown to reduce PON-1 activity,^12^ while diets high in antioxidants can increase PON-1 activity.^11^

On the other hand, LCAT has long been recognised as a key regulator of high-density lipoprotein metabolism; however, the role of this enzyme in human atherogenesis and ASCVD has remained controversial.^13^ Earlier studies reported increases^14,15^ and decreases^3,5^ in plasma LCAT activity and concentrations in patients with ASCVD. Studies have also reported that plasma LCAT concentrations can be regulated by a complex interplay of several factors, such as genetics, hormonal, dietary, inflammatory, medication, and lifestyle factors.^15,16^

Furthermore, research has suggested that there may be a correlation between PON-1 and LCAT concentrations, as both enzymes are involved in lipid metabolism and may be regulated by similar factors. Earlier studies have also linked LCAT and PON-1 to several chronic diseases beyond ASCVD, including diabetes, chronic kidney disease, and Alzheimer’s disease.^17,18,19^

This study assessed the association between reduced plasma LCAT and PON-1 levels and an increased risk of ASCVD, and their significance as potential biomarkers for ASCVD in Nigeria and beyond. This study examined plasma LCAT, PON-1, lipid profile, and anthropometric indices in ASCVD patients.

## Methods

### Ethical considerations

Ethical approval for this study was obtained from the University’s Health Research and Ethics Committee of the Lagos State University Teaching Hospital, Ikeja, Lagos, Nigeria (Reference Number: LREC/06/10/1706). Written informed consent was obtained from each enrollee to participate in the study; all participants were residents of Lagos State, Nigeria. Participants’ anonymity was maintained using a barcode system, and the generated data were treated with the utmost confidentiality. The generated data were used only for this research.

### Study design

The study was a cross-sectional case-control study. The sample size of 138 was determined using the prevalence of a previous study in the region,^20^ based on a 10% prevalence rate of cardiovascular disease in Nigeria.^21^ The study enrolled confirmed ASCVD patients attending the Cardio Clinic and those admitted into the State University Teaching Hospital Emergency Medical Wards (both located at Lagos State University Teaching Hospital, Ikeja, Lagos, Nigeria) between March 2022 and March 2023. A simple random sampling technique was used to select patients diagnosed with ischaemic heart disease (IHD), stroke, hypertensive heart disease, myocardial infarction and congestive heart failure as diagnosed by the attending consultant cardiologist based on the patient’s medical history, clinical presentation, electrocardiographic changes, echocardiographic changes, and biochemical characteristics. For the control group, 50 healthy volunteers with no history of chronic or degenerative diseases were selected from among patients’ relatives and staff at the State University Teaching Hospital. A sample frame was drawn from the controls recruited. A table of random numbers was then used to select the controls that participated in the study.

A standard, structured questionnaire was administered via paper to all participants by the researchers (Online Supplementary Figure 1). The questionnaire was used alongside patients’ medical records to address the general characteristics of all participants and to exclude participants on hormonal therapy, lipid-lowering medications, and those with chronic and degenerative diseases, such as diabetes, cancer, and chronic kidney and liver diseases, as these diseases and medications may alter the outcome of the results in the participants.

**FIGURE 1:**
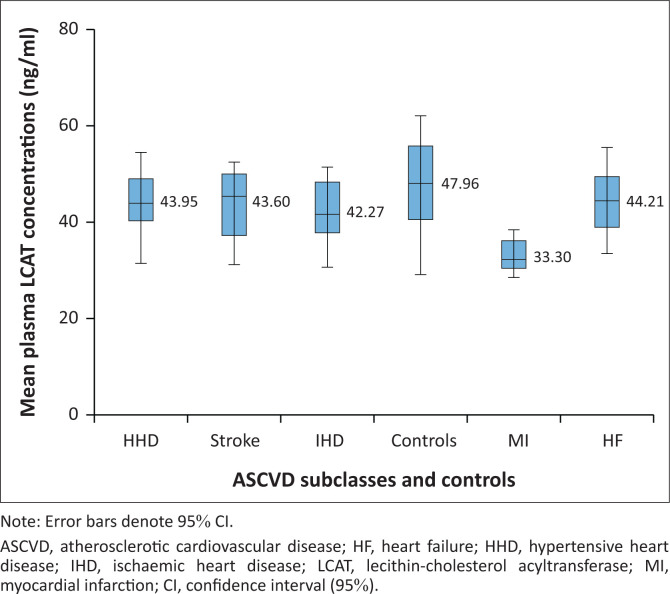
Graded variations in the mean plasma lecithin-cholesterol acyltransferase concentrations in atherosclerotic cardiovascular disease subclasses and controls at Lagos State University Teaching Hospital, Lagos, Nigeria, between March 2022 and March 2023.

### Anthropometry

Anthropometric measurements (weight, height, waist, and hip circumference) were measured using standard procedures. Heights were measured to the nearest centimetre with participants in standing positions, without footwear, using a non-stretchable measuring tape. Each participant’s body weight was measured with light clothing and without shoes on a digital scale (OMRON H BF-514C, Kyoto, Japan). Body mass index (BMI) was then deduced from the participants’ weight (kg) and their height (m); BMI = kg/m^2^. Waist circumference (cm) was measured at the midpoint between the rib cage’s lower border and the hip bones’ iliac crest, measured in the horizontal plane with the participant standing during inspired expiration using a non-stretchable tape. The hip circumference (cm) was taken at the level of the greater trochanter, that is, around the point with the maximum circumference over the buttocks. Both waist and hip circumferences were then used to determine the waist-hip ratio of participants. All participants’ systolic and diastolic blood pressures in millimetres of mercury were measured twice using an OMRON electronic blood pressure monitor (OMRON BP 7250, Kyoto, Japan) and the average was recorded and used in the analyses.

### Blood sample collection

About 7 mL of venous blood were collected by the researchers from patients and controls after an overnight fast of 8 h – 12 h and dispensed into a potassium-ethylene-diamine tetra-acetic acid sample container (1 mg/mL of blood). Each sample was immediately placed in a cold chain until centrifuged at 3000 rpm for 5 min. The plasma was carefully separated from the cells into labelled screw-capped bottles and stored at –20 °C until assayed.

### Biochemical analysis

Lipid profile parameters were assayed using commercially available kits (Randox cholesterol, high-density lipoprotein cholesterol [HDLC], and triglycerides [TG]) from Randox Laboratories Ltd., Crumlin, United Kingdom. The method of Allain et al.^22^ was used for the estimation of total cholesterol (TC) and HDLC, while Bucolo and David’s^23^ method was employed for the estimation of triglycerides. Low-density lipoprotein cholesterol (LDLC) was calculated using Friedewald et al.’s^24^ formulae.


$$\begin{array}{l} \text{LDLC }(\text{mmol/L})=\text{TC }(\text{mmol/L})-\\ \text{‰‰‰‰‰‰‰}(\text{TG}/2.2+\text{HDLC})\;(\text{mmol/L}{)}^{24} \end{array}$$ [Eqn 1]

Non-HDLC (NHDLC) is the sum of all the cholesterol present in each lipoprotein except HDLC, and it is derived by deducting HDLC from the TC of the conventional lipid panel.^25^


$$\text{NHDLC }(\text{mmol/L})=(\text{TC}-\text{HDLC})\;{\left(\text{mmol/L}\right)}^{25}$$ [Eqn 2]

Plasma human PON-1 and LCAT concentrations were determined using the sandwich Enzyme-Linked Immunosorbent Assay method^26^ kit supplied by Elabscience Biotechnology Limited Company, Wuhan, China. Quality control sera were assayed along with the research samples during each batch of tests.

### Data analysis

All results were analysed using Statistical Package for Social Science software version 21 for Windows 10 by IBM Corporation (Chicago, Illinois, United States). The results were expressed as the mean and standard deviation (mean ± s.d.). A Student’s *t*-test was used to compare the statistical difference between the two means, and the differences were regarded as significant at *p* < 0.05. An analysis of variance was used to determine differences within groups, and the differences were regarded as significant at *p* < 0.01. The relationships between LCAT, PON-1, and conventional lipid parameters were assessed using Pearson’s bivariate correlation and were regarded as significant at *p* < 0.01 and *p* < 0.05 (two-tailed).

## Results

### General characteristics of study population

A total of 153 confirmed ASCVD patients (65 men and 88 women) aged between 22 years and 64 years were enrolled in the study. This comprised patients suffering from IHD (*n* = 35), stroke (*n* = 20), hypertensive heart disease (*n* = 43), myocardial infarction (*n* = 15), and congestive heart failure (*n* = 40). A total of 112 patients attended the Cardio Clinic and 41 were patients admitted into the State University Teaching Hospital Emergency Medical Wards. Controls comprised 50 healthy volunteers (22 men and 28 women) aged between 22 years and 64 years. There were statistically significant differences between ASCVD patients compared to controls, with ASCVD patients generally having higher values for mean age, weight, waist circumference, waist-hip ratio, BMI, systolic blood pressure, diastolic blood pressure (all *p* < 0.001), as well as hip circumference (*p* = 0.030) and pulse (*p* = 0.003) (Table 1). When ASCVD male patients were compared to female patients, statistically significant variations were only observed for mean height, waist circumference, waist-hip ratio (all *p* < 0.001), as well as weight (*p* = 0.006) and hip circumference (*p* = 0.013). No significant difference was observed in the mean height of ASCVD patients compared to the controls.

**TABLE 1:** General characteristics, anthropometry, blood pressure, and pulse readings of atherosclerotic cardiovascular disease patients and controls at Lagos State University Teaching Hospital, Lagos, Nigeria, between March 2022 and March 2023.

Variables	ASCVD patients			Controls *N* = 50 (mean ± s.d.)	Post hoc test *p*-value	
	All patients *N* = 153 (mean ± s.d.)	Male patients *n* = 65 (mean ± s.d.)	Female patients *n* = 88 (mean ± s.d.)		ASCVD patients vs controls	ASCVD male vs female patients
Age (years)	52.92 ± 10.24	53.49 ± 9.57	52.22 ± 9.23	46.96 ± 11.05	< 0.001*	0.406
Height (m)	1.64 ± 0.06	1.67 ± 0.80	1.62 ± 0.09	1.64 ± 0.05	0.953	< 0.001**
Weight (kg)	74.05 ± 8.37	76.19 ± 7.98	72.47 ± 8.35	67.74 ± 6.88	< 0.001*	0.006**
Hip circumference (m)	1.04 ± 0.09	1.02 ± 0.05	1.06 ± 0.10	1.01 ± 0.03	0.030*	0.013**
Waist circumference (m)	0.97 ± 0.04	0.95 ± 0.04	0.98 ± 0.04	0.91 ± 0.05	< 0.001*	< 0.001**
Body mass index (kg/m^2^)	27.46 ± 2.60	27.14 ± 2.50	27.69 ± 2.35	25.14 ± 2.04	< 0.001*	0.195
Waist-to-hip ratio	0.98 ± 0.72	0.93 ± 0.08	0.92 ± 0.09	0.89 ± 0.04	< 0.001*	< 0.001**
Systolic blood pressure (mmHg)	132 ± 15.50	132 ± 13.95	132 ± 16.60	119 ± 6.47	< 0.001*	0.929
Diastolic blood pressure (mmHg)	85 ± 12.10	85 ± 12.01	84 ± 12.19	76 ± 6.68	< 0.001*	0.840
Pulse (bpm)	76 ± 10.34	77 ± 9.11	76 ± 11.16	72 ± 2.62	0.003*	0.411

ASCVD, Atherosclerotic cardiovascular disease; bpm, beats per minute; mmHg, millimetres mercury; *n*, number of participants; *p*, level of significance; s.d., standard deviation.

* , values differ significantly between ASCVD patients and controls (*p* < 0.05);

** , values differ significantly between ASCVD male and female patients (*p* < 0.05).

### Lipid profile, lecithin-cholesterol acyltransferase, and paraoxonase-1 in all participants

Atherosclerotic cardiovascular disease patients had statistically significant differences compared to controls with higher values for mean plasma TC, triglycerides, LDLC, and NHDLC (all *p* < 0.001) (Table 2). Conversely, statistically significant differences were observed for mean plasma HDLC, PON-1, and LCAT (all *p* < 0.001) with lower values for ASCVD patients compared to control values.

**TABLE 2:** Lipid profile, lecithin-cholesterol acyltransferase, and paraoxonase-1 of atherosclerotic cardiovascular disease patients and controls at Lagos State University Teaching Hospital, Lagos, Nigeria, between March 2022 and March 2023.

Variables	Cases *N* = 153† (mean ± s.d.)	Controls *N* = 50‡ (mean ± s.d.)	*t*-value	*p*-value
Total cholesterol (mmol/L)	4.87 ± 0.72	3.72 ± 0.42	10.786	< 0.001*
Triglyceride (mmol/L)	1.33 ± 0.36	0.94 ± 0.25	7.075	< 0.001*
High-density lipoprotein cholesterol (mmol/L)	1.06 ± 0.11	1.23 ± 0.10	−9.587	< 0.001*
Low-density lipoprotein cholesterol (mmol/L)	3.20 ± 0.60	2.07 ± 0.39	12.531	< 0.001*
Non-high-density lipoprotein cholesterol (mmol/L)	3.79 ± 0.73	2.92 ± 0.76	7.257	< 0.001*
Lecithin-cholesterol acyltransferase (ng/mL)	42.54 ± 6.67	47.96 ± 8.77	−4.596	< 0.001*
Paraoxonase-1 (ng/mL)	3.81 ± 0.93	6.28 ± 0.87	−16.514	< 0.001*

ASCVD, Atherosclerotic cardiovascular disease; *N*, number of participants; *p*-value, level of significance; s.d., standard deviation; *t*-value, Student’s *t*-test.

* , values differs significantly between ASCVD patients and controls (*p* < 0.05).

† , male patients = 65, female patients = 88;

‡ , male controls = 22, female controls = 28.

### Lipid profile, lecithin-cholesterol acyltransferase, and paraoxonase-1 in atherosclerotic cardiovascular disease subclasses

Statistically significant differences were observed in mean values of plasma TC, triglycerides, LDLC, and NHDLC (all *p* ≤ 0.001) with higher values for subclasses of ASCVD compared with controls (Table 3). On the other hand, statistically significant differences were noted in mean values for plasma HDLC, PON-1, and LCAT (all *p* ≤ 0.001) with lower values for subclasses of ASCVD compared with controls. The largest differences in atherogenic lipids (TC and triglycerides) and lipoproteins (LDLC and NHDLC) were observed among patients with myocardial infarction and IHD. Conversely, these same two ASCVD subclasses also recorded the lowest concentrations of plasma HDLC (anti-atherogenic lipoprotein). A similar trend was observed in the non-conventional lipid-related ASCVD biomarkers (PON-1 and LCAT), where myocardial infarction and IHD patients were noted to have the lowest concentrations of plasma PON-1 and LCAT.

**TABLE 3:** Lipid profile, lecithin-cholesterol acyltransferase, and paraoxonase-1 of different atherosclerotic cardiovascular disease subclasses and controls at Lagos State University Teaching Hospital, Lagos, Nigeria, between March 2022 and March 2023.

Variable	Hypertensive heart disease *N* = 43 (mean ± s.d.)	Ischaemic heart disease *N* = 35 (mean ± s.d.)	Myocardial infarction *N* = 15 (mean ± s.d.)	Heart failure *N* = 40 (mean ± s.d.)	Stroke *N* = 20 (mean ± s.d.)	Controls *N* = 50 (mean ± s.d.)	*F*-value	*p*-value
Total cholesterol (mmol/L)	4.65 ± 0.71	5.29 ± 0.75	5.34 ± 0.55	4.69 ± 0.64	4.63 ± 0.44	3.72 ± 0.42	34.871	< 0.001*
Triglyceride (mmol/L)	1.17 ± 0.31	1.65 ± 0.32	1.63 ± 0.24	1.21 ± 0.31	1.17 ± 0.24	0.94 ± 0.25	31.539	< 0.001*
High-density lipoprotein cholesterol (mmol/L)	1.09 ± 0.13	1.03 ± 0.07	0.99 ± 0.09	1.10 ± 0.11	1.06 ± 0.08	1.23 ± 0.10	23.605	< 0.001*
Low-density lipoprotein cholesterol (mmol/L)	3.04 ± 0.62	3.51 ± 0.62	3.60 ± 0.47	3.06 ± 0.53	2.98 ± 0.38	2.07 ± 0.39	42.070	< 0.001*
Non-high-density lipoprotein cholesterol (mmol/L)	3.58 ± 0.73	4.19 ± 0.80	4.28 ± 0.54	3.64 ± 0.62	3.51 ± 0.45	2.92 ± 0.76	17.519	< 0.001*
Lecithin-cholesterol acyltransferase (ng/mL)	43.95 ± 6.09	42.27 ± 5.93	33.30 ± 3.45	44.21 ± 6.10	43.60 ± 6.09	47.96 ± 8.77	11.399	< 0.001*
Paraoxonase-1 (ng/mL)	4.03 ± 0.86	3.20 ± 0.60	3.25 ± 0.62	4..22 ± 1.08	4.02 ± 0.74	6.28 ± 0.87	71.422	< 0.001*

ASCVD, Atherosclerotic cardiovascular disease; *F*-value, analysis of variance of ASCVD subclasses and controls; *N*, number of participants; *p*, level of significance; s.d., standard deviation.

* , values differ significantly between ASCVD subclasses and controls (*p* ≤ 0.01).

### Association between lecithin-cholesterol acyltransferase, paraoxonase-1, and conventional lipid profiles

A statistically significant, negative correlation was observed between mean body weight and both PON-1 (*r* = -0.246, *p* = 0.002) and LCAT (*r* = -0.236, *p* = 0.001) (Table 4). A strong negative correlation was noted between LCAT and atherogenic LDLC (*r* = -0.213, *p* = 0.004), TC (*r* = -0.197, *p* = 0.007), and NHDLC (*r* = -0.190, *p* = 0.009) among all ASCVD patients. On the other hand, LCAT correlated positively with PON-1 (*r* = 0.151, *p* = 0.031) among ASCVD patients.

**TABLE 4:** Pearson bivariate correlation between lecithin-cholesterol acyltransferase, paraoxonase-1 and conventional lipid profile of atherosclerotic cardiovascular diseases patients at Lagos State University Teaching Hospital, Lagos, Nigeria, between March 2022 and March 2023.

Variable	*r*	*p*-value
Paraoxonase-1 (ng/mL) – weight (kg)	−0.246	0.002**
Lecithin-cholesterol acyltransferase (ng/mL) – weight (kg)	−0.236	0.001**
Lecithin-cholesterol acyltransferase (ng/mL) – low-density lipoprotein cholesterol (mmol/L)	−0.213	0.004**
Lecithin-cholesterol acyltransferase (ng/mL) – non-high-density lipoprotein cholesterol (mmol/L)	−0.190	0.009**
Lecithin-cholesterol acyltransferase (ng/mL) – total cholesterol (mmol/L)	−0.197	0.007**
Lecithin-cholesterol acyltransferase (ng/mL) – paraoxonase-1 (ng/mL)	0.151	0.031*

ASCVD, Atherosclerotic Cardiovascular diseases; *p*, level of significance; *r*, correlation coefficient.

* , correlation is significant at the 0.01 level;

** , correlation is significant at the 0.05 level.

### Variations in lecithin-cholesterol acyltransferase and paraoxonase-1 concentrations in atherosclerotic cardiovascular disease subclasses

**FIGURE 2:**
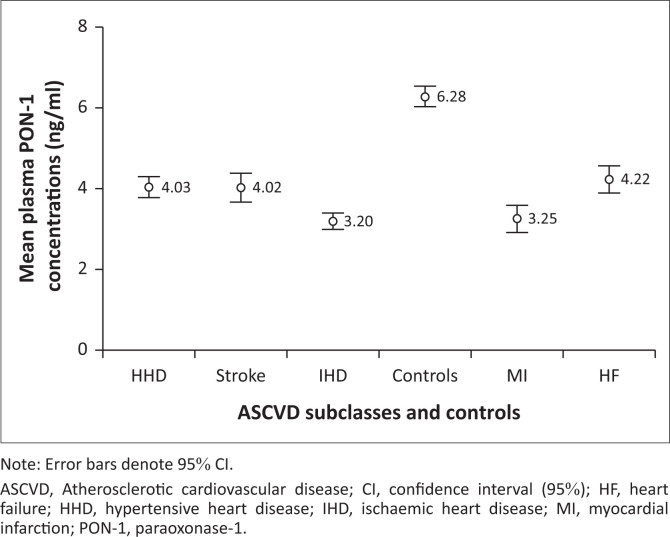
Graded variations in the mean plasma paraoxonase-1 concentrations in atherosclerotic cardiovascular disease subclasses and controls at Lagos State University Teaching Hospital, Lagos, Nigeria, between March 2022 and March 2023.

Graded variations were observed in the mean plasma LCAT concentrations in the various subclasses of ASCVD studied (Figure 1). Myocardial infarction patients had the lowest mean plasma LCAT level. Similarly, graded reductions were also observed in the mean plasma PON-1 concentration in ASCVD subclasses (Figure 2). The lowest mean plasma PON-1 concentration was noted in IHD patients, followed by patients suffering from myocardial infarction.

### Gender and lecithin-cholesterol acyltransferase concentrations

**FIGURE 3:**
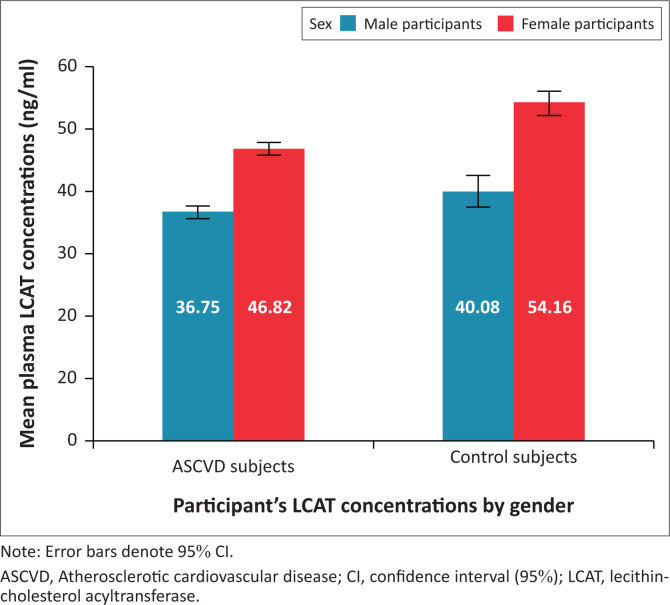
Stratification of lecithin-cholesterol acyltransferase concentrations based on gender in atherosclerotic cardiovascular disease patients and controls at Lagos State University Teaching Hospital, Lagos, Nigeria, between March 2022 and March 2023.

Plasma LCAT concentrations were significantly elevated in female participants compared to their male counterparts (Online Supplementary Table 1). A marked gender discrepancy in LCAT concentrations was also observed among ASCVD patients and controls (Figure 3).

## Discussion

The present study showed that the mean weight, waist circumference, hip circumference, waist-to-hip ratio, BMI, and systolic blood pressure and diastolic blood pressure were significantly higher in ASCVD patients compared to the mean control values. These findings were consistent with previous studies conducted in Europe that reported significant increases in the BMI, waist circumference, waist-to-hip ratio, and blood pressure in ASCVD patients.^27^ Although obesity (BMI > 30 kg/m^2^) was not a distinctive attribute in any of the ASCVD subclasses, earlier studies have shown that the listed anthropometric variables are obesity-related parameters and well-established risk factors for ASCVD.^28^

The present study also reported severe alterations in atherogenic lipids and lipoproteins in ASCVD patients. There were significant increases in plasma TC, triglycerides, LDLC, and NHDLC, while HDLC (anti-atherogenic lipoprotein) was remarkably reduced compared to the control values. In addition, it is worth noting that the lipid profiles of the different subclasses of ASCVD showed significant variations among the different groups assayed.

Notably, the highest increase in atherogenic lipids and lipoproteins (TC, triglycerides, LDLC, and NHDLC) concentrations was observed in myocardial infarction and IHD patients. Conversely, these classes of ASCVD also demonstrated the lowest reduction in the levels of the anti-atherogenic HDLC compared to other groups. These findings were similar to an earlier report by Ebesunun et al.^20^ in southwest Nigeria in 2013, and Hedayatnia et al.^29^ in northeastern Iran in 2020. Both studies reported severe alteration in the atherogenic lipids and lipoproteins in patients suffering from myocardial infarction and IHD compared to other classes of ASCVD. The findings in the present study further confirmed the severity of dyslipidaemia in these classes of patients.

In addition, this study showed a remarkably significant reduction in the mean plasma PON-1 concentration in all the established cases of ASCVD patients compared to the mean control values. This result is consistent with previous studies in other populations worldwide that associated low PON-1 concentration and activity with an increased risk of major cardiovascular events.^6,8^ The reduction in plasma PON-1 concentrations observed in the present study probably suggests impaired antioxidant and anti-inflammatory functions, which may contribute to the progression of atherosclerosis. Oxidised LDLC can accumulate in arterial walls,^30^ leading to the formation of atherosclerotic plaques, and reduced PON-1 activity could further exacerbate this process.

It is also important to note that the lowest reduction in plasma PON-1 concentrations occurred in patients suffering from IHD as well as myocardial infarction. The mean plasma PON-1 concentration in both cases was approximately 50% less than the mean control values. Thus, it suggests the crucial role PON-1 plays in the presentation of IHD and myocardial infarction.

Furthermore, worthy of note is the significant reduction in the mean plasma LCAT concentrations observed in the ASCVD patients compared to the control values. This finding was at variance with some earlier studies that showed no association between LCAT and cardiovascular disease,^14,31^ but on the other hand, it was in agreement with others,^32,33^ which reported reduced LCAT activity and concentration in patients with cardiovascular disease and individuals at high cardiovascular risk.

The reduction in the mean plasma LCAT concentrations observed in ASCVD patients may reflect impaired cholesterol esterification and HDL metabolism. This can lead to reduced HDL functionality and impaired reverse cholesterol transport, a process in which excess cholesterol is transported from peripheral tissues back to the liver for excretion.^13^ Dysfunctional HDL and impaired reverse cholesterol transport are associated with an increased risk of atherosclerosis and ASCVD.^13^

It is noteworthy that the mean plasma LCAT concentrations of the different subclasses of ASCVD showed significant variations among different groups, with the lowest value observed in myocardial infarction patients. This observation also agreed with earlier studies^32,33^ which reported a marked reduction in plasma LCAT activity in patients suffering from myocardial infarction. The marked decrease in LCAT concentrations observed in myocardial infarction patients may have a causal relationship with its aetiology in these patients.

Another important and striking finding in this present study is the marked gender discrepancy in the mean plasma LCAT concentration of the participants studied. Plasma LCAT concentrations were remarkably elevated in the female participants compared to their male counterparts, both in the ASCVD patients and the controls. This finding is consistent with that of earlier studies^14,30^ in other communities around the world that reported higher values of plasma LCAT concentrations in females compared to their male counterparts.

In addition, a significant negative correlation was observed between plasma PON-1, LCAT, and the mean body weight (an established risk factor for ASCVD). A strong inverse correlation observed between LCAT and the atherogenic lipid and lipoproteins (TC, LDLC, and NHDLC) probably emphasised the important role played by LCAT in part in countering the effect of atherogenic lipids and lipoproteins in ASCVD patients.

Furthermore, the present study demonstrated a significant positive correlation between plasma LCAT and PON-1, suggesting that the two lipoprotein-associated enzymes may play synergistic roles on HDL particles to counter the effects of atherosclerosis and ASCVD progression.

### Limitations

One notable limitation of this study is the absence of recent prevalence data on ASCVD in Nigeria that could inform sample size calculations. This limitation was addressed using the available data, and the sample size was adjusted upward. We suggest that future studies may benefit from updated prevalence data on ASCVD.

### Conclusion

The present study demonstrated significant decreases in plasma PON-1 and LCAT levels with severe alterations in atherogenic lipids and lipoproteins in ASCVD patients, especially the subtypes of myocardial infarction and IHD. These observations provide valuable supportive evidence for the interplay of PON-1, LCAT, and lipid metabolism in the pathogenesis of this disease. These findings could potentially lead patients, in part, to develop therapeutic strategies for ASCVD prevention and treatment in future research.
